# A right atrial fat ball: Rare case of cardiac lipoma

**DOI:** 10.1002/ccr3.2335

**Published:** 2019-07-25

**Authors:** Anandbir Singh Bath, Vishal Gupta, Jagadeesh K. Kalavakunta

**Affiliations:** ^1^ Department of Internal Medicine Western Michigan University School of Medicine Kalamazoo MI USA; ^2^ Department of Cardiology Ascension Borgess Heart Institute Kalamazoo MI USA

**Keywords:** benign neoplasms, cardiac MRI, cardiac neoplasms, lipoma

## Abstract

Due to varied presentation, a high index of suspicion is needed for diagnosis of cardiac lipoma. Treatment should only be reserved for symptomatic patients. This case acknowledges the importance of cardiac MRI in making the diagnosis of cardiac lipoma and further delineating the management options available.

A 74‐year‐old asymptomatic man was found to have 3.3 × 2.9 cm right atrial mass on routine transthoracic echocardiogram (Figure 1). On cardiac magnetic resonance imaging (MRI), the mass was confirmed to be a 3.0 × 4.3 × 5.9 cm cardiac lipoma arising from the interatrial septum (Figure 2A‐B). Management of such masses is a therapeutic dilemma. A cardiothoracic surgeon recommended conservative management.

**Figure 1:**
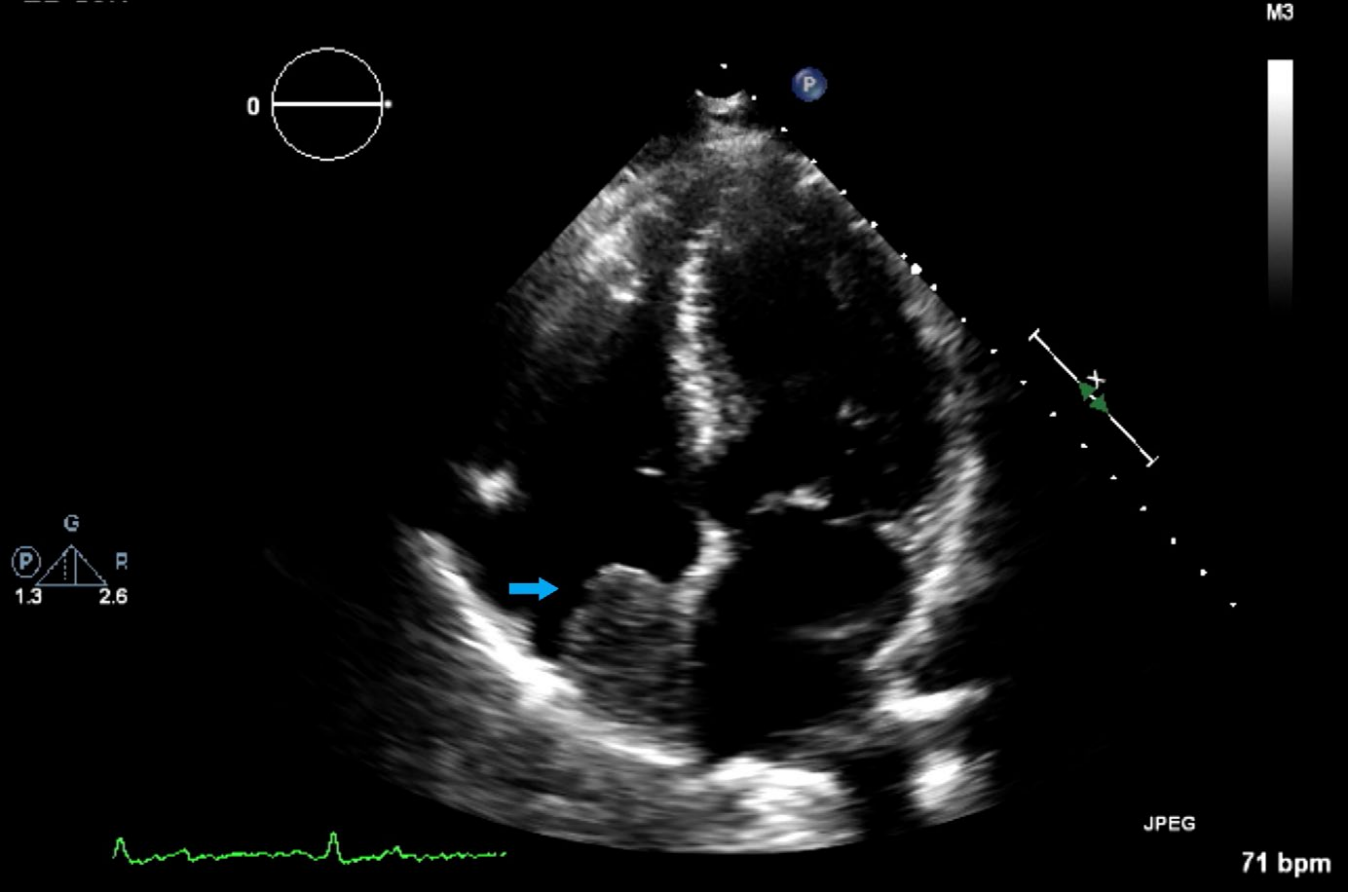
Arrow showing cardiac lipoma in the right atrium on transthoracic echocardiogram

**Figure 2:**
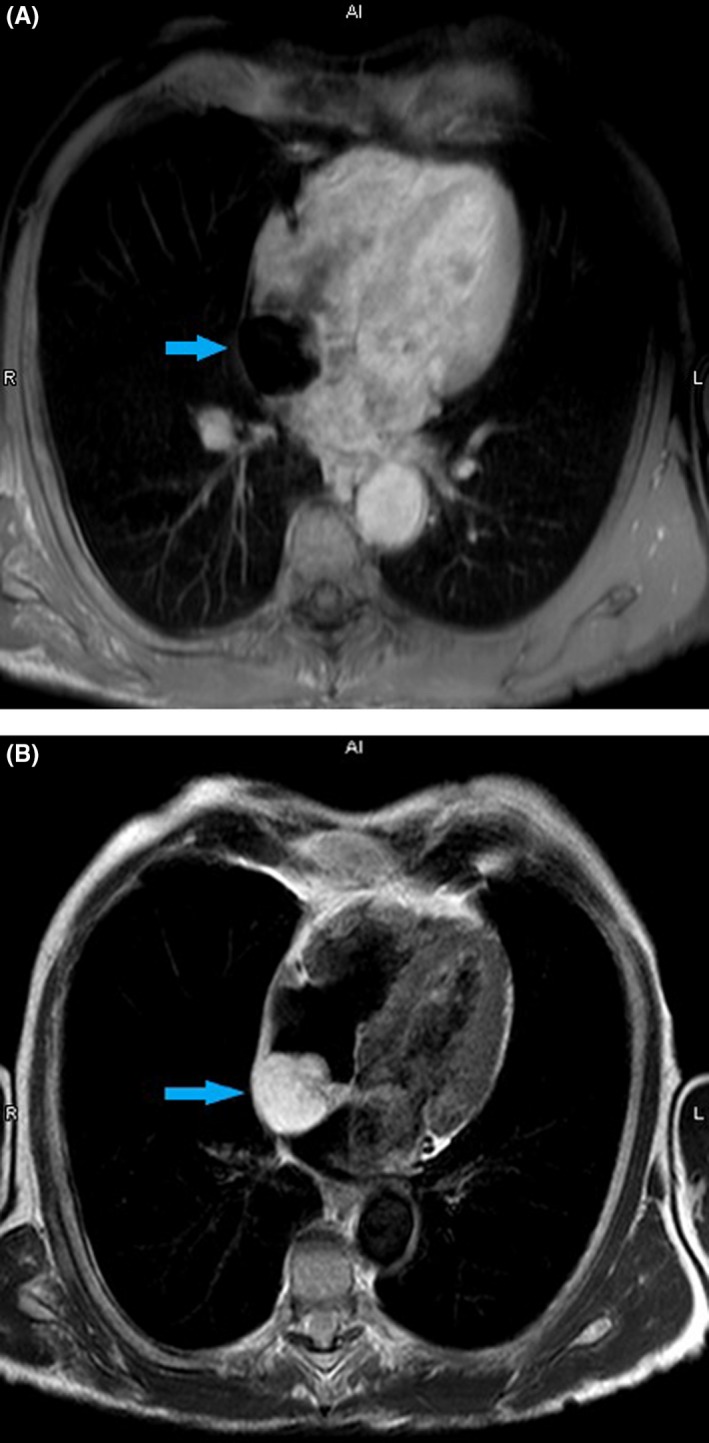
A, Arrow showing cardiac lipoma—T1 weighted sequence in Cardiac MRI. B, Arrow showing cardiac lipoma—T2 weighted sequence in Cardiac MRI

Lipomas are benign encapsulated tumors composed of mature adipocytes commonly originating from the subendocardial and subpericardial layer. They account for 14% of all benign cardiac masses.^1^ Though generally asymptomatic, symptoms include angina, shortness of breath, syncope, arrythmias, and heart failure. Transthoracic echocardiogram has high sensitivity and specificity for detecting cardiac masses, but further delineation needs advanced imaging modalities. Cardiac MRI and CT help in characterization of the tumor.^2^ Revelation of hypersignal in T1 sequences with attenuated images in T2 sequence confirms the diagnosis. Differential diagnosis includes lipomatous hypertrophy of atrial septum, which is an unencapsulated mass of mature and fetal adipocytes.

Complete surgical resection is suggested in symptomatic patients due to high risk of recurrence. Close monitoring is recommended for incidental lipomas. If increasing size is leading to clinical symptoms, then surgery is indicated.

## AUTHOR CONTRIBUTIONS

Anandbir Singh Bath: involved in manuscript preparation and literature search. Vishal Gupta: involved in manuscript reviewing. Jagadeesh K Kalavakunta: involved in manuscript reviewing and editing.
